# Calcium Chloride Modified Alginate Microparticles Formulated by the Spray Drying Process: A Strategy to Prolong the Release of Freely Soluble Drugs

**DOI:** 10.3390/ma11091522

**Published:** 2018-08-24

**Authors:** Marta Szekalska, Katarzyna Sosnowska, Anna Czajkowska-Kośnik, Katarzyna Winnicka

**Affiliations:** Department of Pharmaceutical Technology, Medical University of Białystok, Mickiewicza 2c, 15222 Białystok, Poland; katarzyna.sosnowska@umb.edu.pl (K.S.); anna.czajkowska@umb.edu.pl (A.C.-K.)

**Keywords:** polymer cross-linking, alginate modification, calcium chloride, microparticles, spray drying, prolonged drug release

## Abstract

Alginate (ALG) cross-linking by CaCl_2_ is a promising strategy to obtain modified-release drug delivery systems with mucoadhesive properties. However, current technologies to produce CaCl_2_ cross-linked alginate microparticles possess major disadvantages, such as a poor encapsulation efficiency of water-soluble drugs and a difficulty in controlling the process. Hence, this study presents a novel method that streamlines microparticle production by spray drying; a rapid, continuous, reproducible, and scalable technique enabling obtainment of a product with low moisture content, high drug loading, and a high production yield. To model a freely water-soluble drug, metformin hydrochloride (MF) was selected. It was observed that MF was successfully encapsulated in alginate microparticles cross-linked by CaCl_2_ using a one-step drying process. Modification of ALG provided drug release prolongation—particles obtained from 2% ALG cross-linked by 0.1% CaCl_2_ with a prolonged MF rate of dissolution of up to 12 h. Cross-linking of the ALG microparticles structure by CaCl_2_ decreased the swelling ratio and improved the mucoadhesive properties which were evaluated using porcine stomach mucosa.

## 1. Introduction

Polysaccharides are polymeric carbohydrate molecules commonly exploited in the design of pharmaceutical formulations with prolonged drug release. These polymers possess many advantages such as non-toxicity, wide availability, simplicity to receive, and gelling ability using different cross-linking agents [1]. Sustained release formulations enable a prolonged release profile, which reduces the frequency of drug applications, minimizes side effects, and improves patient’s compliance [2,3]. Dosage forms with mucoadhesive properties provide continuous contact with the mucosal membrane and as a consequence, a prolonged drug residence time and improved drug absorption and bioavailability can be achieved [4,5]. Mucoadhesive microparticles are an example of multi-unit carriers, where the active substance is incorporated in a natural or synthetic polymer matrix. Additionally, microparticles are characterized by a high surface area of drug release and short diffusion pathway, which enables the improvement of the therapeutic efficacy and a reduction of the drug toxicity [6,7].

Sodium alginate (ALG) is a non-toxic polymer which naturally occurs in seaweeds. It may also be synthesized by *Azotobacter* and *Pseudomonas* bacteria [8,9]. ALG is a polysaccharide consisting of β-d-mannuronic and α-l-guluronic acid residues linked by (1–4) glycosidic bonds. ALG is a biocompatible, nonirritant polymer with favorable swelling, gelling, and mucoadhesive properties, thus, it has a wide range of applications in drug delivery technology. ALG hydrogels can be obtained by various cross-linking methods, such as ionic modification (“egg-box” model) based on the binding of cations by the guluronate. In ionic cross-linking, CaCl_2_ is commonly exploited [10,11]. The cross-linking of water-soluble ALG enables an improvement in the polymer stability. Hence, CaCl_2_ cross-linked ALG has been widely examined as a material for pharmaceutical formulations. Additionally, an important feature of CaCl_2_ cross-linking is that the process can be conducted in an aqueous environment at a low temperature, and that both macromolecular and low molecular weight therapeutic agents can be encapsulated [12,13,14,15]. Cross-linked ALG is also utilized in biomedicine—in cell encapsulation or tissue engineering [16,17,18]. The commonly used and well-described method to obtain ALG microparticles cross-linked by CaCl_2_ is the internal gelation by emulsification method [19,20,21,22]. Major disadvantages of microparticles obtained by this technique include poor encapsulation efficiency of water-soluble drugs, low production yield, and organic solvent residues [8,23,24,25].

The spray-drying technique is a useful and valuable method to formulate spherical particles with low moisture content, high drug loading, and high production yield [25,26,27,28]. Therefore, in the present study, the opportunity of using a one-step drying process to formulate modified ALG microparticles was evaluated. As the slow release of active substances with high water solubility is a great challenge in pharmaceutical formulation designing, in the next step the influence of cross-linking on the metformin hydrochloride (MF) release, swelling, and mucoadhesive properties of designed microparticles was determined [2,3]. MF—a biguanide methyl derivative widely used as a first-line drug in non-insulin dependent diabetes mellitus was used as a model of a freely soluble drug [29].

## 2. Materials and Methods 

### 2.1. Materials

Metformin hydrochloride (MF) was a product of Debao Fine Chemical CO (Henan, China). Sodium alginate (ALG) (with viscosity 132.6 mPa∙s of 2% solution), mucin, and gelatin were purchased from Sigma Aldrich (Steinheim, Germany). Potassium dihydrogen phosphate, sodium hydroxide, hydrochloric acid, methanol, propan-1,2-diol, acetonitrile, and calcium chloride were from Chempur (Piekary Śląskie, Poland). Water was purified by osmosis system, Milli-Q Reagent Water System (Billerica, MA, USA). Porcine stomach mucosa was derived from the local veterinary service.

### 2.2. Formulation of CaCl_2_ Modified ALG Microparticles

After preliminary studies, to prepare the microparticles: 2% (*w/w*) ALG, and drug:polymer ratio 2:1 was selected, see Table 1 [30], in addition to 0.5%, 0.1% and 0.05% (*w/w*) CaCl_2_ were applied. In the first step, ALG solutions were prepared and poured into the CaCl_2_ solution, after which their viscosity was determined using a rotational viscometer (Viscotester E Plus—Thermo Haake, Karlsruhe, Germany) [31]. The parameters of the spray-drying process (Mini Spray Dryer B-290, Büchi, Flawil, Switzerland) were set as follows: Flow rate of 4.5 mL/min, spray rate of 37 m^3^/h, and spray flow of 600 L/h. The inlet and outlet temperatures were 115 °C and 46 °C, respectively. As a control, non-modified ALG microparticles (formulation C) were applied.

### 2.3. Evaluation of Microparticles

#### 2.3.1. Shape and Size

To characterize shape and morphology of the microparticles, a scanning electron microscope (SEM) (Hitachi S4200, Tokyo, Japan) was utilized. The size distribution of microparticles suspended in propane-1,2-diol was examined by a Zetasizer NanoZS90 (Malvern Instruments, Malvern, UK) using at least three repetitions for each sample.

#### 2.3.2. High Performance Liquid Chromatography (HPLC) Assay

MF concentration was studied by the HPLC method using an Agilent Technologies 1200 system (Agilent, Waldbronn, Germany) and a Waters Spherisorb^®^ 5.0 μM ODS 4.6 × 250 mm, 5 μm column (Waters Corporation, Milford, MA, USA). As the mobile phase, an acetonitrile: methanol: phosphate buffer pH 3.0 (20:20:60, *v/v*) with a flow rate of 1.0 mL/min was exploited [30].

#### 2.3.3. Drug Encapsulation

To assess MF loading, 20 mg of microparticles was dissolved in 10 mL of phosphate buffer pH 6.8. After 24 h of agitation in a water bath, solutions were filtrated, analyzed by the HPLC method [30], and drug loading (L) was calculated from the expression:

L = Q_m_/W_m_ × 100 (1)

where Q_m_—drug encapsulated in the microparticles, and W_m_—microparticles weight.

Drug encapsulation efficiency (EE) was computed from the formula:

EE = Q_a_/Q_t_ × 100 (2)

where Q_a_—actual drug content, Q_t_—theoretical drug content.

Yield of production (Y) was determined based on the equation:

Y = W_m_/W_t_ × 100 (3)

where W_m_—microparticles weight, W_t_—theoretical calculated drug and polymer weight.

#### 2.3.4. Zeta Potential

Directly after microparticles were suspended in propane-1,2-diol, Zeta potential values were determined by Zetasizer NanoZS90 (Malvern Instruments, Malvern, UK) using Zetasizer Software 6.20.

#### 2.3.5. Swelling Characteristics

The swelling ratio (SR) was tested at 37 ± 1 °C in 0.1 M HCl (pH = 1.2) based on the expression [32]:

SR = W_S_ − W_0_/W_0_ × 100 (4)

where W_0_—microparticles weight, W_S_—swollen microparticles weight.

#### 2.3.6. Mucoadhesiveness

Mucoadhesive properties were evaluated at 37 ± 1 °C by a TA. XT. Plus Texture Analyzer (Stable Micro Systems, Godalming, UK). Gelatin, mucin, and porcine stomach mucosa were used as mucoadhesive layers [33]. Process parameters, chosen during preliminary tests, were as follows: Pretest speed 0.5 mm/s, test speed 0.1 m/s, contact time 180 s, post-test speed 0.1 mm/s, applied force 1 N. Mucoadhesiveness was expressed as the detachment force (F_max_) and the work of mucoadhesion (W_ad_).

### 2.4. MF Dissolution

MF dissolution from microparticle formulations (in the amount equivalent to 500 mg of MF) was performed at 37 ± 1 °C in a basket apparatus (Erweka Dissolution Tester Type DT 600HH, Heusenstamm, Germany) in 500 mL of 0.1 M HCl (pH = 1.2) [34]. MF concentration in the release medium was studied by the HPLC technique (as described in the point 2.3.2. HPLC analysis).

### 2.5. Mathematical Modeling of the MF Release Profile 

To explain the drug release mechanism, data obtained from MF release tests were studied under different mathematical models [35,36]. Zero order kinetic:

F = k × t(5)

first order kinetic:

lnF = k × t(6)

Higuchi model:

F = kt^1/2^ (7)

Korsmeyer-Peppas model:

F = kt^n^ (8)

Hixson-Crowell model:

1 − (1 − F)^1/3^ = kt (9)

where F—the fraction of released drug, k—the constant connected with release, and t—the time. 

### 2.6. Index of Similarity and Dissimilarity

To compare release profiles of designed microparticles, a model utilizing a difference factor *f*_1_ and similarity factor *f*_2_ was applied. The difference index *f*_1_ was calculated by the formula:

*f*_1_ = {(∑ = 1n|R_t_ − T_t_|) (∑ = 1nR_t_)} × 100 (10)

where n—number of samples, R_t_ and T_t_—data of drug dissolution of control and test sample at the same time (t). The difference index *f*_1_ expresses the percent variation between release from the studied and control sample. The similarity index *f*_2_ indicates the potential release similarity and it is calculated by the following equation:

*f*_2_ = 50 × Log {[1 + (1/n)∑ = 1n(R_t_ − T_t_) × 2] − 0.5 × 100} (11)

*f*_2_ between 50–100 indicates release profiles similarity between samples [37,38,39].

### 2.7. Differential Scanning Calorimetry (DSC)

Measurements were performed using an automatic thermal analyzer system (DSC TEQ2000, TA Instruments, New Castle, DE, USA). Samples (in the amount of 5 mg) were placed in aluminum pans and heated in the range 25–280 °C under a 20 mL/min nitrogen flow [40].

### 2.8. Statistics

Data were assessed by Statistica 10.0 (StatSoft, Tulsa, OK, USA) using one-way analysis of variance (ANOVA) or a Kruskal-Wallis test. Obtained results were presented as the mean and standard deviation.

## 3. Results and Discussion

**Figure 1:**
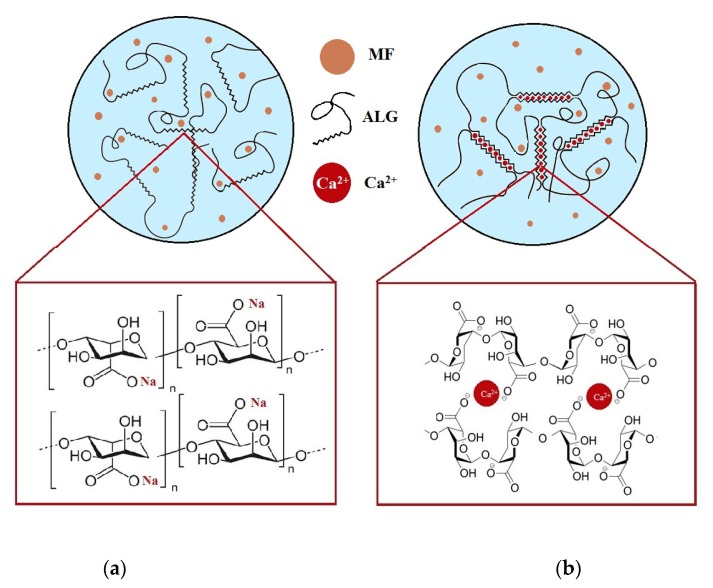
Structure of non-modified (**a**) and CaCl_2_ cross-linked (**b**) ALG microparticles containing MF.

ALG is a polymeric material commonly utilized in the design of sustained drug delivery systems via its in situ gelation when in contact with the acidic stomach environment. As a result of hydrogen bonding, ALG alternates into insoluble alginic acid, which controls water penetration and prevents matrix disintegration. Additionally, to improve the mechanical strength of ALG and to reduce the solubility in water, a cross-linking process by ionic interactions with divalent cations such as Ca^2+^ can be performed [41]. The gelation process is a result of the linkage by Ca^2+^ of guluronate regions in one ALG backbone with a similar region in another ALG molecule, which creates a cross-linked structure. Application of a cross-linking agent leads to a reduction in the solubility of the polymer matrix under acidic conditions, which could be an effective approach to sustain the release of freely water-soluble drugs [42,43,44]. A Schematic structure of the non-modified and CaCl_2_ cross-linked formulation with MF is presented in Figure 1.

### 3.1. Microparticles Characteristics

To receive CaCl_2_ modified ALG microparticles, 2% ALG and 0.05% or 0.1% CaCl_2_ solutions were chosen for the one-step spray-drying process. A 2% ALG solution modified with 0.5% CaCl_2_ possessed a high viscosity and its spray drying was limited, see Table 2.

**Figure 2:**
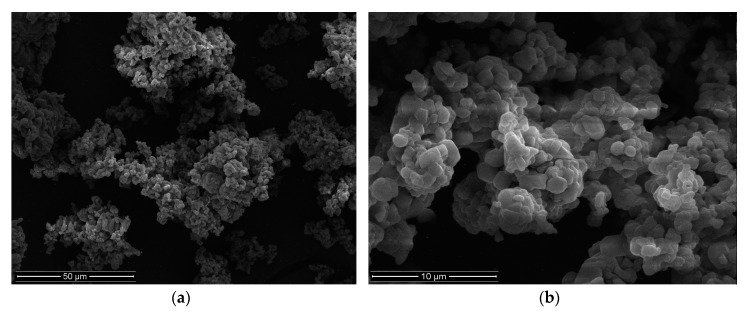
Images of microparticles CA1 (**a**) ×2000, (**b**) ×10000.

The quality evaluation of obtained microparticles included analysis of particle size, MF percent loading, drug encapsulation efficiency, production yield, and Zeta potential, see Table 3. The morphology of microparticles formulation CA1 is presented in Figure 2.

Obtained data revealed that the cross-linking agent induced only a slight increase in the mean diameter of the microparticles (from 3.0 ± 1.6 µm in formulation C to 3.5 ± 1.2 µm in CA1) and ALG modification by CaCl_2_ cross-linking resulted in a small decline in production yield. Interestingly, ALG modification did not influence MF loading percentage in the microparticles, and all formulations were characterized by a drug loading of above 70%, as shown in Table 3. Additionally, it was observed that the cross-linking process resulted in a slight reduction in the encapsulation efficiency (from 113.4 ± 2.3% in formulation C to 91.5 ± 2.1% in CA1).

ALG is an anionic polymer with a negative charge, but positively charged MF and Ca^2+^ ions changed the Zeta potential [45,46,47] and designed microparticles possessed a positive charge, as shown in Table 3.

### 3.2. Swelling and Mucoadhesive Properties

The ALG swelling ability is a peculiar parameter affecting mucoadhesiveness and drug release. In contact with moisture, ALG begins to hydrate and swell, and as a consequence, forms a hydrogel which regulates the influx of the aqueous medium and drug dissolution [44]. As a result of gelling, the water influx is decreased; thus, the drug release is prolonged. Dissolution of freely water-soluble drugs from hydrophilic carriers is generally regulated by the drug’s diffusive properties via the hydrogel matrix. Therefore, the swelling properties might significantly affect the drug release profiles [47].

**Figure 3:**
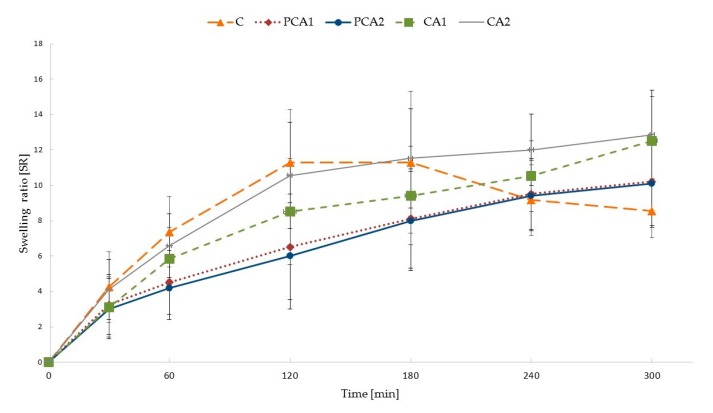
Swelling characteristics of non-modified C and cross-linked formulations PCA1, PCA2, CA1, and CA2.

The swelling behavior of designed microparticles was examined in an acidic environment and presented as the swelling ratio (SR), see Figure 3. At an acidic pH, ALG carboxylate groups on the surface of the microparticles are protonated, and water-insoluble alginic acid is formed, which impedes the penetration of fluid into the deeper layers of the particle matrix [48,49]. After contact with this medium, non-modified ALG microparticles take up the fluid, which penetrates into the matrix and leads to an increase of SR to 180 min. Formation of insoluble alginic acid leads to a limitation of swelling, and even shrinkage of particles, which was observed as a decreased SR of formulation C at 240 min. CaCl_2_ cross-linked microparticles exhibited lower SR values than non-modified formulation C, and a linear increase up to 300 min was observed. The lower amount of swelling in cross-linked microparticles is the result of insoluble calcium alginate formation upon contact with the acidic medium, which leads to a lower water inflow into the matrix [48,49,50]. It was also found that microparticles CA1 and CA2 possessed higher values of SR, compared to the placebo formulations (PCA1 and PCA2), which is due to the occurrence of freely soluble molecules of MF and a faster influx of the aqueous medium into the microparticle structure.

**Figure 4:**
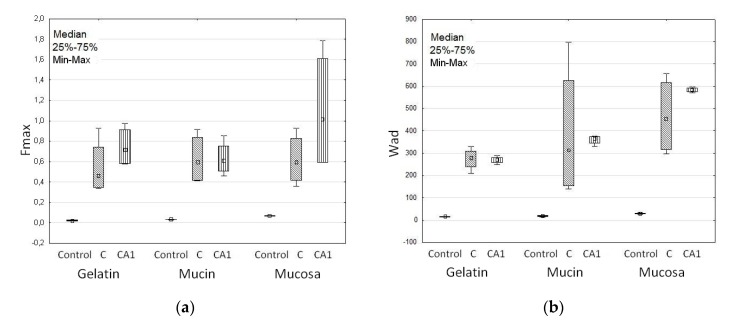
Mucoadhesive features: (**a**) Maximum detachment force (F_max_) and (**b**) work of adhesion (W_ad_) of non-modified formulation C, cross-linked formulation CA1 and cellulose paper (Control) (median; n = 6).

The swelling process extends the contact time of the drug carrier with the mucosa and improves drug bioavailability. After contact with the hydrated mucus, ALG absorbs moisture and polymer chains with groups containing hydrogen bonds are relaxed [51]. This process, initiating deep contact of the polymer with the mucus layer enables the linkage of microparticles with mucosa and leads to the phenomenon of mucoadhesion [51,52]. Mucoadhesive properties of designed microparticles are presented in Table 4. All formulations adhered to tested materials, and mucoadhesiveness was considerably (*p* < 0.05) influenced by the type of adhesive layer, and the presence and concentration of CaCl_2_. When a gelatin disc and mucosa gel were used, similar values of F_max_ and W_ad_ were noted, as shown in Table 4. In the case of porcine stomach mucosa, the highest F_max_ values—median from 0.6 N (C) to 1.1 N (CA1) and W_ad_ values from 454.8 μJ (C) to 583.2 μJ (CA1)—were observed, see Table 4 and Figure 4. Porcine stomach mucosa is a valuable model of the adhesive layer due to its similarity to human mucosa in terms of histology, ultrastructure, and composition, and it can be used to reflect the behavior of dosage forms in vivo [53]. It was demonstrated that in an acidic environment CaCl_2_ cross-linking reduces ALG interaction with the mucous membrane as a result of poor swelling ability [54,55,56]. However, in this study improvement of the mucoadhesieve properties of CaCl_2_ cross-linked ALG microparticles was observed. This fact can be explained by the presence of Ca^2+^ ions, which interact with the negatively charged mucin [57,58]. An increase in the positive charge of the polymer leads to better interactions with sialic acid and other anionic groups present in mucin and the formation of additional bonds with the negatively charged membrane [59,60]. With a higher concentration of cross-linking agent, increased work of adhesion was observed. Interestingly, freely water-soluble MF did not influence the mucoadhesive properties of the formulations.

### 3.3. MF Dissolution

**Figure 5:**
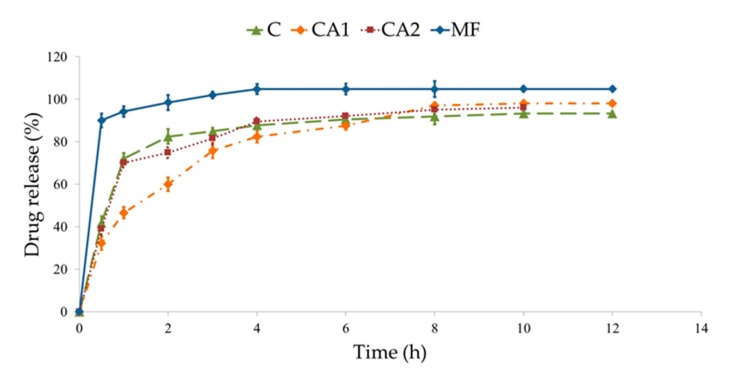
MF dissolution from non-modified microparticles C, cross-linked CA1, CA2, and commercial product used as a control (MF).

Dissolution of the active substance from microparticles depends on the drug’s water solubility, influx of the medium into the structure of the dosage form, and polymer swelling [49,50]. In drug dissolution from all microparticles, a burst effect, caused by rapid dissolution of freely soluble MF bound at the microparticle surface, was noted and can be seen in Figure 5. Additionally, it was observed that MF was released faster from non-modified microparticles C (82.5 ± 3.6% MF was released after 2 h). This fact is related to the higher SR properties of the unmodified polymer in an acidic environment, which expedites the influx of the aqueous medium into the matrix. Modification of the ALG structure by CaCl_2_ affected MF dissolution—the release behavior from formulation CA2 (obtained with 0.05% of CaCl_2_) was comparable to non-modified microparticles C, but formulation CA1 (with 0.1% of CaCl_2_) significantly prolonged MF release. It was shown that in formulation CA1 60.1 ± 3.8%, MF was released in the first 2 h and sustained to 12 h, whereby it reached 97.5 ± 2.7%. Ca^2+^ cross-linking leads to a more stable and more intact structure, improves the mechanical resistance of the polymeric network, and reduces its swelling ability [61].

MF dissolution was also analyzed by different mathematical equations, see Table 5. It was shown that from designed microparticles, MF was released according to first-order kinetics. In the model of Highuchi, where the best-fit curve with a high R^2^ was observed, it was proved that MF release was diffusion controlled. In the model of Korsmeyer-Peppas, values of index *n* were from 0.08 to 0.12 and confirmed diffusion-dependent MF dissolution. In comparison to formulation C, the Hixson-Crowell model exhibited a better linear relationship with a regression index from 0.94 to 0.96, indicating that the dominant mechanism of MF release from modified microparticle formulations is diffusion coupled with erosion determined by the presence of a cross-linking agent [62].

During the design of oral formulations, it is crucial to explain the drug release mechanism, but also to evaluate if the development of a new technological process affects the modification of the drug release profile compared to the conventional dosage form. Therefore, the similarity and dissimilarity factors were determined to measure the similarity of MF dissolution profiles and to express the potential product similarity [63]. The difference and similarity indices *f*_1_ and *f*_2_, following the international (FDA, EMA) guidelines for the dissolution profile comparison were used [64,65]. According to these guidelines, release profiles are comparable if they possess a value of *f*_1_ in the range 0–15 and *f*_2_ in the range 50–100 [66].

MF release profiles of formulations CA1 and CA2 were compared with non-modified formulation C and a commercial product with non-modified MF release was used as a reference sample. When a non-modified tablet containing MF was used as a control, it was observed that all designed formulations have a similarity factor of <50 and difference factor of >15. Therefore, it was concluded that both non-modified microparticles and formulations modified by CaCl_2_ were characterized by different drug release profiles. To assess the impact of a cross-linking agent on the MF dissolution process, an independent model approach was applied. It was found that in formulation CA2, values of difference and similarity factors were 4.05 and 72.27, respectively, which indicates a similar MF dissolution profile to formulation C. On the other hand, the formulation CA1 was characterized by the difference factor 17.55 and similarity factor 42.88, which indicates that CaCl_2_ concentration affected MF dissolution.

### 3.4. Differential Scanning Calorimetry (DSC)

**Figure 6:**
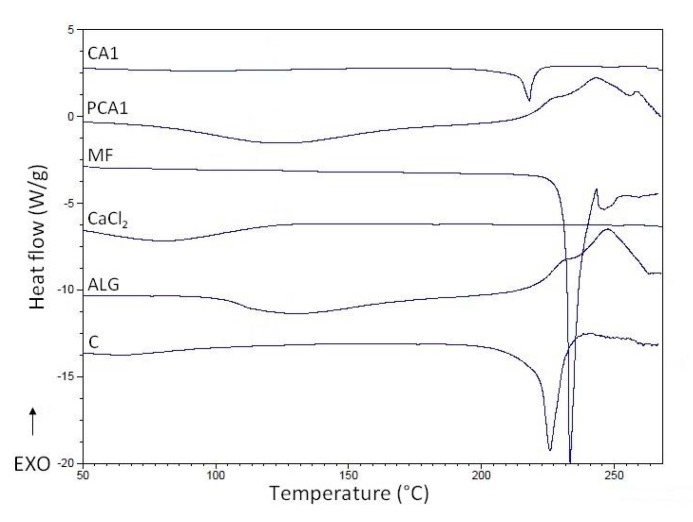
Differential scanning calorimetry (DSC) curves of pure sodium alginate (ALG), metformin (MF), CaCl_2_, non-modified microparticles C, microparticles PCA1, and CA1.

To evaluate the thermal characteristics of the materials and excipients used, DSC technology was utilized [67]. The MF thermogram exhibits a sharp endothermic peak at 233.02 °C, which corresponds to the melting point of pure MF, see Figure 6. The DSC curves of ALG and PCA1 were similar, and broad endothermal peaks between 100 °C and 150 °C were observed, which indicates the loss of water content in the polymer. Additionally, a sharp exothermic peak related to ALG decomposition at 248 °C was observed. The thermogram of PCA1 shows an exothermic peak registered at 252.6 °C, which might suggest an interaction between CaCl_2_ and ALG. On the other hand, the MF peak demonstrated a slight decrease in the melting temperature in the CA1 formulation (218.13 °C) in comparison to MF (226.57 °C), which is probably as a result of interactions between MF and the polymer, and formation of prim polymer matrix [68].

## 4. Conclusions

Drug solubility exerts an evident impact on the mechanism and release kinetics. The rapid dissolution of active compounds characterized by being freely soluble in water is one of the main drawbacks when designing pharmaceutical formulations. To prolong the release of MF used as a model freely water-soluble drug, physical modification of ALG microparticles by CaCl_2_ cross-linking using a novel one-step drying process was applied. The developed method involves considerably fewer unit operations than traditional emulsification techniques. The in vitro drug release data showed a significant difference among cross-linked and non-cross-linked formulations. This study demonstrates that CaCl_2_ cross-linked ALG microparticles can be successfully used to prolong MF release. Additionally, it was observed that ALG cross-linking using CaCl_2_ decreased the swelling ratio and improved the mucoadhesive properties of microparticles evaluated using porcine stomach mucosa.

## Figures and Tables

**Table 1:** Components of designed microparticles formulations.

Formulation	ALG ^1^ (%)	MF ^2^:ALG ^1^ Ratio	CaCl_2_ (%)
C	2	2:1	–
PCA1	2	–	0.1
PCA2	2	–	0.05
CA1	2	2:1	0.1
CA2	2	2:1	0.05

^1^ Sodium alginate, ^2^ Metformin hydrochloride.

**Table 2:** Viscosity of CaCl_2_ cross-linked ALG solutions.

Solution	Viscosity (mPa∙s) ^1^
2% ALG	132.6 ± 2.7
0.5% CaCl_2_ + 2% ALG	4700.4 ± 14.5
0.1% CaCl_2_ + 2% ALG	1600.6 ± 12.4
0.05% CaCl_2_ + 2% ALG	791.7 ± 5.3

^1^ n = 3.

**Table 3:** Quality evaluation of non-modified microparticles C and cross-linked microparticles CA1 and CA2 containing MF.

Microparticles	Zeta Potential (mV)	Production Yield (%)	Encapsulation Efficiency (%)	Loading Percentage (%)	Particle Size (µm)
C	−1.3 ± 0.7	61.7 ± 2.1	113.4 ± 2.3	75.6 ± 1.5	3.0 ± 1.6
CA1	2.6 ± 0.4	57.1 ± 1.6	91.5 ± 2.1	77.1 ± 1.7	3.5 ± 1.2
CA2	2.6 ± 0.9	56.1 ± 2.4	93.9 ± 2.7	73.9 ± 3.4	3.4 ± 1.4

**Table 4:** Mucoadhesive properties of designed microparticles.

Formulation	Kind of Adhesive Material					
	Gelatine Disc		Mucin Gel		Porcine Stomach Mucosa	
	F_max_ (N) ^1^	W_ad_ (µJ) ^2^	F_max_ (N) ^1^	W_ad_ (µJ) ^2^	F_max_ (N) ^1^	W_ad_ (µJ) ^2^
Control ^3^	0.02 ± 0.01	15.2 ± 0.7	0.03 ± 0.01	18.1 ± 3.5	0.07 ± 0.01	29.4 ± 3.3
C	0.5 ± 0.2	283.3 ± 51.2	0.6 ± 0.3	342.3 ± 29.3	0.6 ± 0.2	467.5 ± 17.4
PCA1	0.7 ± 0.1	291.3 ± 14.4	0.5 ± 0.2	362.9 ± 18.8	1.3 ± 0.3	519.7 ± 16.9
PCA2	0.6 ± 0.2	272.3 ± 13.1	0.5 ± 0.1	353.4 ± 21.4	1.2 ± 0.1	504.6 ± 21.3
CA1	0.7 ± 0.2	269.4 ± 14.1	0.6 ± 0.2	359.2 ± 18.5	1.1 ± 0.7	583.4 ± 15.7
CA2	0.6 ± 0.1	254.6 ± 16.7	0.7 ± 0.3	347.1 ± 32.1	1.3 ± 0.2	500.5 ± 13.5

^1^ Maximum detachment force, ^2^ Work of adhesion, ^3^ Cellulose paper.

**Table 5:** Mathematical characteristics of MF dissolution.

	Mathematical Model										
Formulation	Zero Order		First Order		Highuchi		Korsmeyer-Peppas			Hixson-Crowell	
	R^2^	K	R^2^	K	R^2^	K	R^2^	K	*n*	R^2^	K
C	0.52	4.62	0.73	0.21	0.66	18.76	0.59	0.28	0.12	0.65	0.19
CA1	0.86	1.68	0.96	0.55	0.95	29.37	0.88	0.34	0.08	0.94	0.58
CA2	0.92	1.67	0.98	0.17	0.97	22.98	0.94	0.31	0.08	0.96	0.18

R^2^: correlation coefficient, K: dissolution constant, n: the dissolution index
